# Trends in Infant Mortality in United States: A Brief Study of the Southeastern States from 2005–2009

**DOI:** 10.3390/ijerph120504908

**Published:** 2015-05-06

**Authors:** Xiaojia He, Luma Akil, Winfred G. Aker, Huey-Min Hwang, Hafiz A. Ahmad

**Affiliations:** 1 Department of Biology, Jackson State University, 1400 JR Lynch Street, Jackson, MS 39056, USA; E-Mails: xiaojia.he@hotmail.com (X.H.); lumaakil@hotmail.com (L.A.); winfredaker@gmail.com (W.G.A.); huey-min.hwang@jsums.edu (H.-M.H.); 2 Department of Computer Science, Jackson State University, 1400 JR Lynch Street, Jackson, MS 39056, USA

**Keywords:** infant mortality, public health, southeastern states, socioeconomic status, Alabama, Louisiana, Mississippi, Georgia, Florida

## Abstract

While overall infant mortality rates have declined over the past several decades, the Southeastern states have remained the leading states in high infant death in the United States. In this study, we studied the differences in infant mortality in the southeastern United States from 2005 through 2009 according to mother’s characteristics (age of mother, marital status, maternal race, maternal education), birth characteristics (month when maternal prenatal care began, birth weight), and infant’s characteristics (age of infant at death). This paper illustrates the significance level of each characteristic of mothers and infants, as well as socioeconomic factors that contribute to significant infant mortality that impacts subgroups within the US population. Descriptive statistics and analysis of variance studies were performed and presented. Statistical analysis of the contribution of causes of infant death to infant mortality at the national and state level was elaborated. Data suggest that mothers with no prenatal care had a very high overall infant death rate (5281.83 and 4262.16 deaths per 100,000 births in Mississippi and Louisiana, respectively, whereas the US average was 3074.82 deaths (*p* < 0.01)). It is suggested that better education and living quality should be available and improved for the residents in Alabama, Louisiana, and Mississippi.

## 1. Introduction

The significance of infant mortality as an important indicator of a nation's health status and well-being has been well documented in social and biomedical research. Globally, the infant mortality rate decreased from 6300 deaths per 100,000 live births in 1990 to 3500 deaths per 100,000 live births in 2012 [1]. Correspondingly, the infant mortality rate of the United States (US) declined from approximately 10,000 infant deaths per 100,000 live births in 1900, to 689 infant deaths per 100,000 live births in 2000 [2]. However, the US rate is still largely above those of most other developed countries, which has attracted attention among researchers and public policy makers.

Infant mortality is associated with a variety of factors such as maternal health, socioeconomic conditions, and public health practices. The high US infant mortality has some association with and may be attributable in large part to disparities in socioeconomic status which in turn are associated with race and ethnicity. Considerable differences in socioeconomic status and resulting financial disempowerment may adversely affect food security and nutrition, education, and health care in local and regional communities. Thus, the long-term pattern of mortality in the US has shown inconsistency for socioeconomic and demographic subgroups of the population as well as causes of infant death. Clearly, divisive social issues have led the Southeastern states to having the most serious poverty problems in the country (for this study, the Southeastern states include: Alabama, Florida, Georgia, Mississippi, and Louisiana). For instance, Mississippi has the highest poverty rate in the US according to the American Community Survey, showing 17.40% of families living below the poverty level in 2011 [3], compared to 11.7% for the entire country. The Southeastern states’ average infant mortality rate has been approximately 10 deaths per 1000 live births in recent years. According to the Mississippi State Department of Health [4], and Alabama Department of Public Health [5], 8.8 and 8.9 babies per 1000 born in 2012 died before their first birthday, respectively. This issue makes the infant mortality rate in Mississippi more comparable to those of developing countries such as Chile (8.0 per 1000 live births), Lebanon (8.0 per 1000 live births), and Oman (10 per 1000 live births) than that of the US (6.0 per 1000 live births), according to latest estimates (2012) from the World Health Organization (WHO) [6]. Coincidently, teenage pregnancy is also more prevalent in the Southeast than in most other parts of the country, according to the latest Centers for Disease Control and Prevention (CDC) data. For instance, Mississippi ranked the highest for teen birth rate in 2010, with 55 out of 1000 women compared to a national average of 34.2 [7].

A comprehensive investigation and analysis of past trends of infant mortality is not only critical in developing effective public healthcare programs and policies, but also vital for future health planning. However, no systematic effort has yet been established to bring together a thorough assessment of recent courses and differentials in the Southeastern states’ infant mortality in relation to those important sociodemographic variables. Hence, the purpose of this paper: to analyze long term trends and differentials in infant mortality in five southeastern states from 2005 to 2009 by location, cause of death, mother's characteristics, birth characteristics, and infant's characteristics in the following states Alabama (AL), Florida (FL), Georgia (GA), Louisiana (LA), and Mississippi (MS). These states were selected based on the geographical location and their resembling socioeconomic status, and then these states were compared with the US average.

## 2. Methods

This is a retrospective study of data on infant deaths and proportionality of socioeconomic factors collected from the CDC, the American Community Survey, the Department of Commerce, and the US Census Bureau. Only the records of infant death infants under one year of age, as verified by official birth certificates, were collected. We used four main data categories encompassing nine statistical variables (Location: southeastern US; cause of death; Mother’s Characteristics: age of mother, marital status, maternal race, maternal education; Birth Characteristics: month when maternal prenatal care began, birth weight; Infant's Characteristics: age of infant at death) for our analysis, all based on linked live birth–infant death cohort files. We used the 2005–2009 birth cohorts (n = 25,229,455). The latest version of the International Statistical Classification of Diseases and Related Health Problems (ICD) was used for the description of causes of infant death. Data for Month Prenatal Care Began, and Maternal Education were not available or comparable for all selected states in the later period of investigation (2007–2009). Data are presented as mean ± (standard deviation, SD). Descriptive statistics and analysis of variance were performed using SAS-PC version 9.2 (SAS Institute, Cary, NC, USA).

## 3. Results

### 3.1. Location: Overall Trends in Infant Mortality

The infant mortality in the five Southeastern states indicated a downward trend from 2005 to 2009 (Figure S1). The decline in the rate during this period was quite impressive, with the average decline being 3.1%, 2.33%, 2.66%, and 3.05% per year, respectively, for Alabama, Georgia, Louisiana, and Mississippi, whereas it was only 1.17% and 1.73% per year for Florida and the US, respectively. Notably, as indicated in Table S1, all five southeastern states except Florida were significantly different from the US average (*p* < 0.05).

**Table 1:** Infant mortality and poverty level in the Southeastern US from 2005–2009.

Year	State	Infant Mortality (per 100,000)	Ranking	Poverty Level (Percentage of People in Poverty)	Ranking
2005–2009	Alabama	923.80 ± 63.59 *	3/50	15.28 ± 2.08 *	8/50
2005–2009	Florida	715.28 ± 16.28	20/50	11.78 ± 2.44	22/50
2005–2009	Georgia	789.36 ± 31.86 *	8/50	13.64 ± 2.25 *	12/50
2005–2009	Louisiana	936.62 ± 50.57 *	2/50	16.74 ± 1.57 *	2/50
2005–2009	Mississippi	1040.25 ± 63.75 *	1/50	19.46 ± 2.47 *	1/50
2005–2009	US	665.98 ± 17.51	NA	12.10 ± 1.98	NA

* *p* < 0.05, compared to the US.

As shown in Table 1, statistical analysis of infant mortality indicated that Mississippi and Alabama were significantly different from all but Louisiana (*p* < 0.05). Louisiana was significantly different from Georgia and Florida (*p* < 0.05). Georgia was significantly from all but Florida (*p* < 0.05). In addition, the difference in the poverty rate between Mississippi and all the other four states were significant (*p* < 0.05).

### 3.2. Cause of Deaths

Current ICD-10 categories were used to represent the underlying cause of death from the death certificates. All the major causes of infant death in the Southeastern US, 2005 to 2009, are listed in Figure S2. The six leading causes are: P07.2 (Extreme immaturity), R95 (Sudden infant death syndrome), R99 (Other ill-defined and unspecified causes of mortality), P07.3 (Other preterm infants), W75 (Accidental suffocation and strangulation in bed), P22.0 (Respiratory distress syndrome of newborn), as shown in Table S1, and their significance level is shown in Table S2.

### 3.3. Mother’s Characteristics: Age of Mother, Marital Status, Maternal Race, and Maternal Education

**Figure 1:**
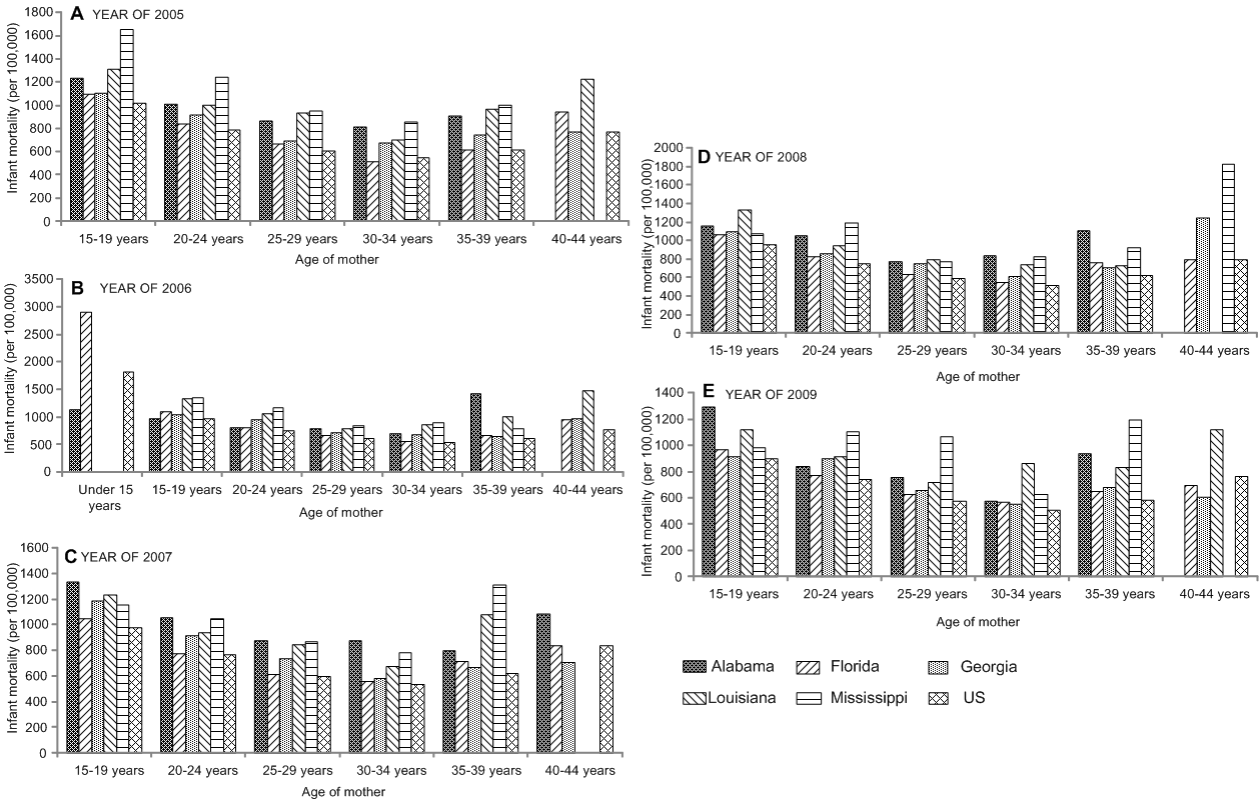
Analysis of variance shows the significant difference in infant mortality by the age of mother in the Southeastern US in years 2005 (**A**) to 2009 (**E**).

Infant mortality rates were highest for mothers in the youngest and oldest age groups (Table S3). The infant mortality rate in the entire US for mothers aged less than 15 years, was 1585.19 infant deaths per 100,000 live births, approximately three times the rates for mothers aged 25–29 years, 30–34 years, and 35–39 years, the age groups of the lowest risk (*p* < 0.05). The significance of infant mortality and age of mother in 2005 to 2009 is shown in Figure 1.

As shown in Table S3, the infant mortality rate for the entire country for mothers aged 45–49 years and 50–54 years was 1163.69 and 1104.65, respectively, approximately twice the rate for mothers in the three age groups at lowest risk.

**Figure 2:**
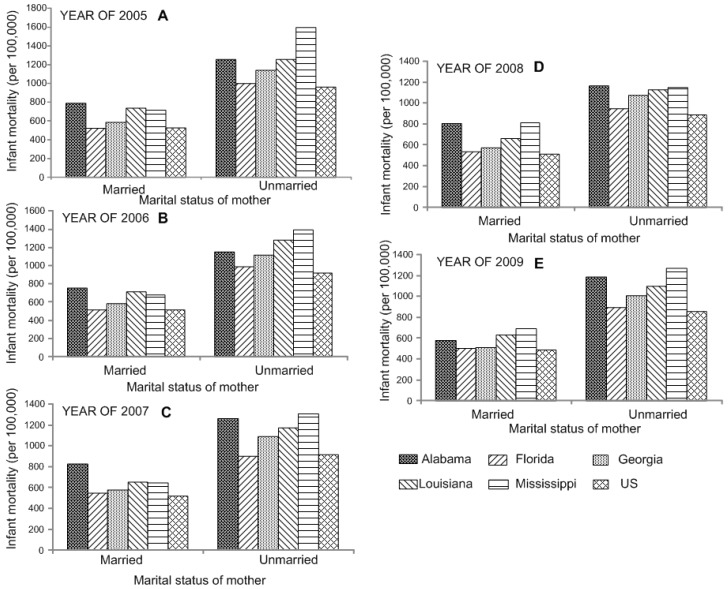
Analysis of variance shows the significant difference in infant mortality by the marital status of mother in the Southeastern US in years 2005 (**A**) to 2009 (**E**).

In addition, the fates of babies born of unmarried mothers were significantly different from that of those born to married ones, with rates almost twice as high (*p* < 0.05, Tables S5 and S6). Differences in infant mortality and marital status in years 2005 to 2009 are shown in Figure 2.

**Figure 3:**
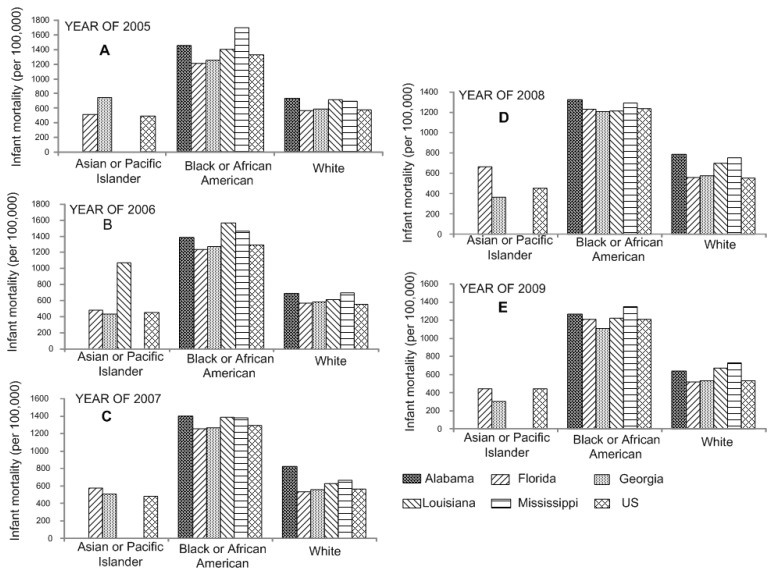
Analysis of variance shows the significant difference in infant mortality by the maternal race of mother in the Southeastern US from 2005 (**A**) to 2009 (**E**).

CDC data for 2005 to 2009, show nationwide infant mortality among black Americans above 10 per 1000 live births (Tables S7 and S8). The difference between infant mortality and maternal race from 2005 to 2009 is shown in Figure 3. Besides, the results indicated that mothers with only 9–11 years of education have the highest infant death rate. For instance, the average infant mortality over five years (2005–2009) was 1630.58 deaths per 100,000 live births in Mississippi (Tables S9 and S10). State comparison for infant mortality based on maternal education from 2005 to 2009 is shown in Figure S3.

### 3.4. Birth Characteristics: Month When Maternal Prenatal Care Began, and Birth Weight

Analysis of variance shows that mothers with no prenatal care had a high overall infant death rate (Figure S4 and Table 2).

**Table 2:** Differences in infant mortality by the month of prenatal care began in the Southeastern US from 2005 to 2009.

Month Prenatal Care Began	Average Infant Mortality (per 100,000) (2005–2009)					
	Alabama	Florida	Georgia	Louisiana	Mississippi	US
No prenatal care	3891.23 ± 1258.15	3607.83 ± 109.01	3314.24 ± 827.48	4262.16 ± 1492.23	5281.83 ± 231.53 *	3074.82 ± 505.11
1st month	893.38 ± 117.67	811.68 ± 60.22	666.79 ± 40.66	929.34 ± 69.34	935.60 ± 1.35	762.40 ± 83.20
2nd month	824.49 ± 46.12	553.09 ± 31.50	583.27 ± 119.41	890.63 ± 143.02	1028.82 ± 125.00	540.92 ± 40.07
3rd month	913.96 ± 31.36	561.68 ± 5.03	739.16 ± 106.19	889.18 ± 102.66	997.71 ± 64.32	533.57 ± 37.40
4th month	935.49 ± 62.49	571.24 ± 36.49	776.03 ± 107.61	1098.92 ± 214.20	1313.59 ± 138.71	611.74 ± 42.24
5th month	811.39 ± 222.35	718.12 ± 67.83	840.30 ± 149.83	1319.22 ± 51.66	1179.05 ± 193.38	709.32 ± 39.65
6th month	914.63	599.15 ± 73.28	720.61 ± 99.71	1146.26 ± 7.88	1516.92	635.48 ± 48.66
7th month	NA	531.54 ± 52.06	610.04 ± 121.25	NA	NA	571.98 ± 61.32
8th month	NA	422.98 ± 32.23	NA	NA	NA	546.13 ± 35.28

* *p* < 0.05, compared to the US.

For example, the rates were 5281.83 and 4262.16 deaths per 100,000 births in Mississippi and Louisiana, respectively, whereas the US average was 3074.82 deaths per 100,000 births (*p* < 0.01, Table 2 and Table S11). Moreover, our data analysis suggested that 85% or more of babies with birth weight less than 499 grams would die (Table 3 and Table S12). Our data show that birth weights less than 2500 grams could lead to a relatively high infant death rate, normally higher than 10 deaths per 1000 births (Figure S5).

**Table 3:** Differences in infant mortality *vs*. birth weight in the southeastern us from 2005 to 2009.

Birth Weight (Grams)	Average Infant Mortality (per 100,000) (2005–2009)					
	Alabama	Florida	Georgia	Louisiana	Mississippi	US
499 or less	89,431.77 ± 2522.16	91,231.54 ± 1966.83 *	78,770.35 ± 4335.20 *	56,749.46 ± 31,943.64 *	88,613.34 ± 3311.23	85,692.42 ± 804.39
500–999	30,173.41 ± 2185.68	30,804.51 ± 1753.09	31,030.00 ± 2398.76	27,112.22 ± 3112.62	33,228.92 ± 2308.14	29,506.30 ± 1056.98
1000–1499	5239.12 ± 711.72	5921.19 ± 622.88	6074.62 ± 375.23	6250.05 ± 831.95	5582.69 ± 575.18	5708.09 ± 87.22
1500–1999	2958.55 ± 607.55	2577.66 ± 121.08	2752.05 ± 284.92	3012.60 ± 712.23	3019.64 ± 944.97	2649.00 ± 81.25
2000–2499	1208.561 ± 51.78	1054.42 ± 51.59	1030.13 ± 143.86	1084.49 ± 124.18	1393.16 ± 209.31	1051.68 ± 30.78
2500–2999	499.06 ± 58.17	396.97 ± 21.88	460.67 ± 52.26	530.08 ± 8.67	582.02 ± 104.14	407.20 ± 16.00
3000–3499	278.75 ± 38.29	203.05 ± 11.79	222.49 ± 25.60	314.86 ± 53.77	299.88 ± 41.51	210.95 ± 2.92
3500–3999	213.94 ± 31.71	134.70 ± 9.04	156.39 ± 21.34	219.14 ± 30.70	234.20 ± 66.33	147.29 ± 3.08
4000–4499	NA	143.59 ± 24.46	177.32 ± 42.32	NA	NA	138.21 ± 3.62

* *p* < 0.05, compared to the US.

### 3.5. Infant’s Characteristics: Age of Infant at Death

Our data analysis indicated that the US neonatal and postneonatal mortality rates declined by 7.93% and 4.54%, respectively, from 2005 to 2009 (Tables S13 and S14).

**Figure 4:**
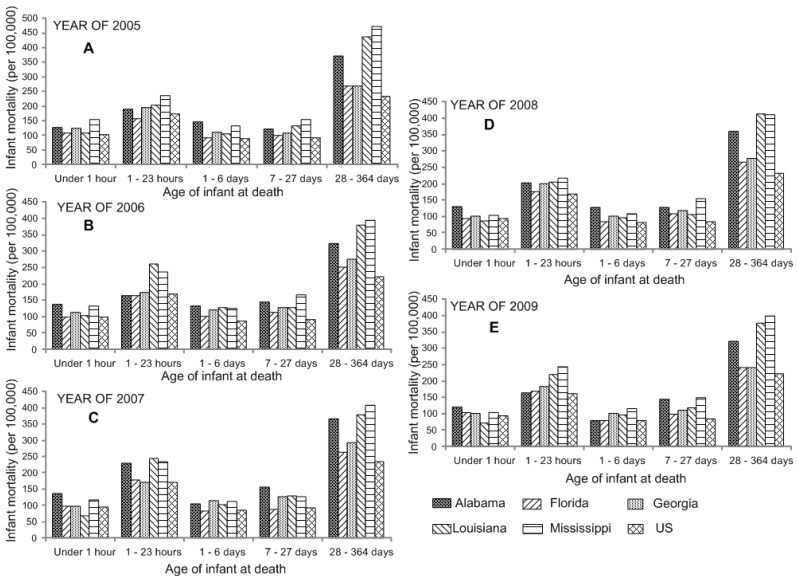
Analysis of variance shows the significant difference in infant mortality by the age of infant at death in the Southeastern US from 2005 (**A**) to 2009 (**E**).

The mortality data analysis also indicated that all the southeastern states have higher neonatal and postneonatal mortality compared with US average; particularly, Alabama, Louisiana, Georgia, and Mississippi were significantly different from the US average of post-neonatal deaths (Figure 4).

## 4. Discussion

### 4.1. Cause of Deaths

Six leading causes of death were revealed by the data analysis as shown in Table S2, and together they accounted for 57% of all infant deaths in the US also in 2010, according to CDC National Center for Health Statistics reports [8]. High death rates among infants so affected, suggest the crucial role of prematurity in infant mortality. Premature birth is recognized as one of the top causes of death in infants worldwide [9]. Considerable risk for short-term and long-term impairments to an individual’s abilities is associated with premature birth. Although extensive efforts to prevent such birth have been made, its prevalence has not been significantly reduced yet [10].

The second leading cause of death among infants is Sudden Infant Death Syndrome (SIDS). According to the US CDC, approximately four thousand infant deaths in the US each year have no obvious cause [11]. Reports from the US CDC have pointed out that, although the overall national rate of SIDS has decreased by approximately 50% since 1990, the rates for non-Hispanic black and American Indian/Alaska Native infants remain high above those for the rest of the population. For instance, SIDS as the cause of infant death in Alabama increased from 38.05 to 94.44 deaths per 100,000 births, in the years 2005 to 2009. Although the rate of SIDS has been declining in Mississippi, it still remains above 100 deaths per 100,000 births. Thus, reducing the risk of SIDS remains a high public health priority and this study is a contributory effort to provide the predictive analytics to effect a level of management.

Yet another sub-group of sudden, unexpected infant deaths (SUIDs) comprises those caused by accidental suffocation and strangulation in bed (ASSB). Couches, poor-fitting crib mattresses, and infant beds filled with clutter are the most frequent sites/causes of the infant deaths by these modes. They caused an average of 60.19 deaths per 100,000 births in Mississippi from 2005 to 2009, while causing commensurately high death rates in Florida and Louisiana as well. Noticeably, the cause of ASSB for infant mortality increased from 25.19 to 32.52 deaths per 100,000 births in Florida during the selected five year period. In addition, Shapiro-Mendoza and coworkers reported that infant mortality rates attributable to ASSB have quadrupled in US since 1984 [12]. Furthermore, recent evidence showed that the decline in SIDS was offset by an increase in ASSB [13]. However, it has been suggested that the procedures for classifying and reporting SUIDs should be modified. Arguably though, increased knowledge of the epidemiology of ASSB deaths must precede any such modifications and any investigations of prevention.

Adding to the list of causes of infant mortality is infant respiratory distress syndrome (IRDS). IRDS is a common syndrome in premature infants caused by developmental immaturity and insufficiency of surfactant production in the lungs. Our data indicated that IRDS is one of the leading causes of neonatal death resulting from respiratory distress, which agrees with existing reports [14]. Rodriguez also suggested that it is associated with the production of surfactant related proteins, the health condition of mother, and the gestational age [14]. Although IRDS plays a major role in infant death statistics, its prevalence decreased in the period 2005 to 2009 for all five southeastern states and the entire US, revealing a positive sign.

According to our results, Louisiana and Mississippi were significantly different from the US average in the first two leading causes, as shown in Table S1. It is clear that extreme immaturity and SIDS are the major problems accounting for the high infant death rates in these two states (Table S2).

### 4.2. Mother’s Characteristics

It is clear that teenage mothers, and mothers aged 40 and older have the highest infant mortality rates. In addition, there is a clear pattern in the 40–44 age group, in these states which differ from the US average with higher infant death rates (Table S4). It was also reported that there was a strong correlation between young maternal age and high infant mortality and between young maternal age and a high prevalence of low birth weight [15].

Marital status is also one of the factors that may be causally correlated with infant mortality. It is suggested that the increased risk of infant mortality is closely associated with single motherhood. Alabama, Louisiana, and Mississippi, exhibited significant higher death rates than the US (*p* < 0.05). In addition, among white mothers, age, education level and receipt of prenatal care all show significant interactions with marital status; the increased risks of infant mortality attributed to unmarried motherhood are concentrated among subgroups usually thought to be at lower risks [16]. Statistical analysis reveals that the infant mortality rate for Black or African-Americans in the southeastern states is higher than that of other ethnic groups, and actually about twice that for Whites, Asians, and Pacific Islanders. It accounts largely for the high mortality rate in those states with higher percentages of minorities, specifically, Mississippi, Louisiana, Alabama, and Georgia.

Furthermore, the racial disparity is consistent in the Southeastern states, where the rates in Alabama, Louisiana, and Mississippi differed significantly from the nation as a whole (*p* < 0.05). In Mississippi, 40% of infants were born to black women, which raised the infant mortality rate of the state as a whole. Additionally, although most black women give birth at term, on average, black women are about 50% more likely to have a premature baby compared to white women [17]. The reasons for the difference remain unknown and are an area for the further research. However, it has been reported that prematurity/low birth weight is the leading cause of death among black infants [18].

This study also examined inequalities in infant mortality in the Southeastern states in relation to maternal educational level. The overall death rate is more than twice the rate for mothers with more than 16 years of education, the group with the lowest infant death rate. This scheme is prevalent throughout these Southeastern states, leading to the inequalities in infant mortality. Three of the five states studied, Alabama, Louisiana, and Mississippi, were significantly different from the US national average, based on our statistics analysis (*p* < 0.05). In substantiation of the education–infant mortality connection, this pattern also holds true for other countries; infant mortality is lowest in the highest educational group and increases in the lower educational groups [19].

### 4.3. Birth Characteristics

Prenatal care refers to the health services that pregnant women receive before a baby’s birth. Having regular monitoring inspection, potential diseases that may endanger the mother or baby can be discovered and treated in a timely manner. In addition, proper prenatal care can provide balanced nutrition for both mother and baby, and foster stable mental health. It is clear that infant death rates increase dramatically with the continuing postponement of the beginning of maternal prenatal care. The infant death rate for mothers with prenatal care beginning from the first month was only one-fifth of the rate for those with no prenatal care.

Furthermore, the initiation and fulfillment of prenatal care are affected by maternal age. Only one-third of mothers under the age of 15, and about half of those aged 15–19 tend to receive prenatal care in the first trimester, indicating young mothers were least likely to seek and receive regular prenatal care [20]. On the contrary, more than 70% of those aged 25 years and older initiated early and timely prenatal care. This is also associated with their tendency to have sufficient education. Prenatal care differentials in infant mortality rates may reflect differences in income, educational levels, access to health care, and health insurance, which are closely related to the age of mother and their independency. Quick and coworkers reported that low birth weight and infant mortality rate were 1.5 to 5 times greater than those with early, frequent care [21]. Because of this, it is important that pregnant women not only begin prenatal care early, but also receive continuous care throughout their pregnancy.

### 4.4. Infant’s Characteristics

Age of infant at death include calculations for two age-specific categories: neonatal deaths and post-neonatal deaths. The neonatal death rate is calculated as the number of infant deaths that occur between 0–27 days of life divided by the number of live births, multiplied by 100,000. The post-neonatal death rate is calculated as the number of infant deaths that occur from 28 days to under one year of life, divided by the number of live births, multiplied by 100,000. Notably, the extremely high post-neonatal mortality in Alabama (348.32 ± 24.10 deaths per 100,000 live births), Mississippi (416.72 ± 31.67 deaths per 100,000 live births), and Louisiana (396.93 ± 27.36 deaths per 100,000 live births) significantly suppressed the overall trend in US (228.01 ± 5.78 deaths per 100,000 live births). It is suggested that post-neonatal mortality is generally related to SIDS, the second leading cause for infant mortality [22]. Noticeably, post-neonatal mortality is closely related to race. According to the report published by U.S. Department of Health and Human Services in 2011 [20], the post-neonatal mortality was 451 deaths per 100,000 live births for Black and Hispanic women, while it was only 184 and 182 deaths per 100,000 live births for non-Hispanic White and Hispanic women, respectively.

## 5. Prospects for the Future and Policy Considerations

Although the rate of infant mortality has been declining and the positive changes have been achieved in controlling the health of new born babies over the past decade, there is still work that needs to be done in the decades ahead. All the Southeastern states, especially Alabama, Louisiana, and Mississippi have much room for improvement of the health and well-being of women, infants and families. Infant mortality still remains at high levels compared to the other regions in the US. Support for early and continuous maternal prenatal care and education are urgent public concerns. Sufficient education could lead to higher family income, better health care, and ultimately benefit our next generation.

As the African-American population of the Southeastern states increases, it becomes increasingly essential that health care providers and policymakers increase their awareness and pay more attention to the cultural background of regional residents, increasing education and training in these aspects of health care delivery. In addition, collaboration between the private and governmental care organizations to improve health care should be enhanced to help mothers and babies remaining in the care systems. State, county, and local governments must address basic mental health and social services needs of the low-income women and families. Fulfilling those needs is essential for creating a healthier future, and a long term agenda for those actions must be identified and instituted in the coming decades.

## 6. Limitations of the Study

A limitation of the present study is that some data is not available from CDC database. For instance, data for Month Prenatal Care Began (Figure 4 and Table 2 and Table S11), and Maternal Education (Figure S3) were not available or comparable for all selected states in the later period of investigation (2007–2009). Another limitation of this study is that we mainly focus on descriptive analysis and the analysis of variance. Future work will pay more attention to the correlation coefficients among all variables with a multivariable approach.

## 7. Conclusions

Age of mother, marital status, maternal race, maternal education, month when maternal prenatal care began, and birth weight play the leading roles in determining the infant death rate in the US, despite the improvement observed during the selected five years. It is most crucial for the high infant death rates in the Southeastern states, Alabama, Louisiana, and Mississippi, particularly. The association between poverty reduction and infant mortality provides an overall view of socioeconomic influence and a gauge of the impact of life-threatening factors. In order to provide a more comprehensive review of their association, more socioeconomic and cultural factors need to be analyzed. It is critical to educate mothers in the necessity to seek prenatal care. Women, with adequate education, could avoid teenage pregnancy and seek appropriate health care. A better socioeconomic status with education could sustain women’s good health prior to becoming pregnant, and ensure proper infant care. It is also critical that effective infant and maternal health monitoring and information systems have the capacity to provide a full spectrum of perinatal outcomes.

## 8. Ethical Approval

No ethical approval is required. No human subjects were directly involved in this research. No identifiable information can be generated through the data used in this research. All the data were secondary obtained from various governmental sites. This research relies exclusively on publicly available information legally accessible to the public and appropriately protected by law through data guardians.

## Supplementary Files

Supplementary File 1
