# Supplementary material for: Trends in Infant Mortality in United States: A Brief Study of the Southeastern States from 2005–2009
Source: Int J Environ Res Public Health. 2015 May 6;12(5):4908–20. doi: 10.3390/ijerph120504908 (PMC4454945; doi:10.3390/ijerph120504908)
Supplement: Supplementary File 1 [file ijerph-12-04908-s001.pdf]

Supplementary Information

Trends in Infant Mortality in United States: A Brief Study of the Southeastern States from 2005–2009

Table S1. Six leading causes for infant mortality in the southeastern states and entire country from 2005 to 2009.

Death Code (ICD-10 Codes)	Cause of Death	Average Infant Mortality (per 100,000) (2005–2009)					
		**Alabama**	**Florida**	**Georgia**	**Louisiana**	**Mississippi**	**US**
P07.2	Extreme immaturity	117.04 ± 16.59	78.15 ± 5.27	95.96 ± 10.33	156.16 ± 14.93 *	154.84 ± 27.20 *	84.48 ± 1.37
R95	Sudden infant death syndrome	70.62 ± 26.62	37.29 ± 3.12	88.96 ± 12.81	126.45 ± 23.36 *	142.04 ± 43.03 *	54.98 ± 1.26
R99	Other ill-defined and unspecified causes of mortality	65.31 ± 20.39 *	30.46 ± 8.23	14.37 ± 8.52	35.13 ± 21.86	35.15 ± 9.36	26.58 ± 2.47
P07.3	Other preterm infants	37.43 ± 18.28	27.42 ± 3.12	39.69 ± 5.91	31.00 ± 13.34	31.98 ± 7.25	26.20 ± 0.53
W75	Accidental suffocation and strangulation in bed	16.97	28.10 ± 4.33	12.48 ± 1.21	25.23 ± 3.89	60.19 ± 24.08 *	15.04 ± 1.92
P22.0	Respiratory distress syndrome of newborn	33.86 ± 9.39	18.52 ± 4.68	29.06 ± 9.01	21.77 ± 4.02	25.64 ± 3.29	15.85 ± 2.45

* $p < 0.05$, compared to the US.

Table S2. Comparison of significance level of six leading causes for infant mortality in southeastern states.

Death Code (ICD-10 Codes)	Significance Level									
	**AL-FL**	**AL-GA**	**AL-LA**	**AL-MS**	**FL-GA**	**FL-LA**	**FL-MS**	**GA-LA**	**GA-MS**	**LA-MS**
P07.2	$p < 0.05$	NS	$p < 0.05$	$p < 0.05$	NS	$p < 0.05$	$p < 0.05$	$p < 0.05$	$p < 0.05$	NS
R95	NS	NS	$p < 0.05$	$p < 0.05$	$p < 0.05$	$p < 0.05$	$p < 0.05$	$p < 0.05$	$p < 0.05$	NS
R99	NS	$p < 0.05$	NS	NS	$p < 0.05$	NS	NS	NS	NS	NS
P07.3	NS	NS	NS	NS	NS	NS	NS	NS	NS	NS
W75	NS	NS	NS	NS	NS	NS	NS	NS	$P<0.05$	NS
P22.0	NS	NS	NS	NS	NS	NS	NS	NS	NS	NS

Table S3. Infant mortality and age of mother in the southeastern states and the entire country from 2005 to 2009.

Age of Mother	Average Infant Mortality (per 100,000) (2005–2009)					
	**Alabama**	**Florida**	**Georgia**	**Louisiana**	**Mississippi**	**US**
Under 15 years	NA	2898.55 *	NA	NA	NA	1585.19 ± 144.87
15–19 years	1230.82 ± 84.90 *	1051.73 ± 52.30 *	1066.96 ± 100.13 *	1265.36 ± 91.27 *	1238.47 ± 266.84 *	961.11 ± 43.44
20–24 years	981.86 ± 88.43	800.54 ± 28.04	907.00 ± 128.81	973.02 ± 60.16	1148.18 ± 76.62 *	760.37 ± 16.43
25–29 years	812.11 ± 55.45	635.97 ± 21.34	709.95 ± 162.10	814.23 ± 77.87	899.41 ± 112.93 *	591.53 ± 11.51
30–34 years	772.65 ± 117.85	545.31 ± 22.06	615.97 ± 156.32	766.48 ± 88.39	794.68 ± 103.22	527.81 ± 16.02
35–39 years	885.00 ± 154.70 *	675.01 ± 59.07	686.74 ± 41.39	920.89 ± 139.24 *	1042.49 ± 210.04 *	609.61 ± 16.01
40–44 years	1255.23 ± 236.30 *	841.39144.71	856.36 ± 254.49	1349.24 ± 178.19 *	1824.82*	783.60 ± 31.10
45–49 years	NA	NA	NA	NA	NA	1163.69 ± 155.53
50–54 years	NA	NA	NA	NA	NA	1104.65 ± 112.53

* $p < 0.05$, compared to the US.

Table S4. Comparison of significance in the correlation between the age of mother and infant mortality in the southeastern states.

Age of Mother	Significance Level									
	**AL-FL**	**AL-GA**	**AL-LA**	**AL-MS**	**FL-GA**	**FL-LA**	**FL-MS**	**GA-LA**	**GA-MS**	**LA-MS**
Under 15 years	NA	NA	NA	NA	NA	NA	NA	NA	NA	NA
15–19 years	NS	NS	NS	NS	NS	NS	NS	NS	NS	NS
20–24 years	NS	NS	NS	NS	NS	NS	$P < 0.05$	NS	NS	NS
25–29 years	NS	NS	NS	NS	NS	NS	NS	NS	NS	NS
30–34 years	NS	NS	NS	NS	NS	NS	NS	NS	NS	NS
35–39 years	NS	NS	NS	NS	NS	NS	$P < 0.05$	NS	$P < 0.05$	NS
40–44 years	$p < 0.05$	$p < 0.05$	NS	$p < 0.05$	NS	$p < 0.05$	$p < 0.05$	$p < 0.05$	$p < 0.05$	$p < 0.05$

Table S5. Infant mortality and marital status in the southeastern states and the entire country from 2005 to 2009.

Year	State	Average Infant Mortality (per 100,000)	
		Married	Unmarried
2005–2009	Alabama	749.65 ± 99.77 *	1204.28 ± 52.81 *
2005–2009	Florida	524.51 ± 15.57	944.72 ± 48.70
2005–2009	Georgia	563.45 31.88	1083.70 ± 50.76 *
2005–2009	Louisiana	677.06 ± 43.23 *	1186.36 ± 81.02 *
2005–2009	Mississippi	707.18 ± 63.06 *	1341.70 ± 164.46 *
2005–2009	US	509.24 ± 14.05	908.35 ± 38.52

* $p < 0.05$, compared to the US.

Table S6. Comparison of significance in the correlation between marital status and infant mortality in the southeastern states.

Marital Status	Significance Level									
	AL-FL	AL-GA	AL-LA	AL-MS	FL-GA	FL-LA	FL-MS	GA-LA	GA-MS	LA-MS
Married	$p < 0.05$	$p < 0.05$	NS	NS	NS	$p < 0.05$	$p < 0.05$	$p < 0.05$	$p < 0.05$	NS
Unmarried	$p < 0.05$	$p < 0.05$	NS	$p < 0.05$	$p < 0.05$	$p < 0.05$	$p < 0.05$	$p < 0.05$	$p < 0.05$	$p < 0.05$

Table S7. Infant mortality with respect to race in the southeastern us from 2005 to 2009.

States	Year	Race	Demographics	Infant Mortality (per 100,000)	Differences Compare to Black Race	Significance Compare to Black Race ($\alpha = 0.05$)
Alabama	2005–2009	Black or African American	26.5%	1367.87 ± 74.72		
	2005–2009	White	70.0%	735.30 ± 72.62	186%	$p < 0.05$
Florida	2005–2009	Asian or Pacific Islander	2.7%	535.45 ± 87.41	230%	$p < 0.05$
	2005–2009	Black or African American	78.3%	1228.94 ± 19.02		
	2005–2009	White	16.6%	548.34 ± 22.58	224%	$p < 0.05$
Georgia	2005–2009	Asian or Pacific Islander	3.5%	469.81 ± 172.40	260%	$p < 0.05$
	2005–2009	Black or African American	31.2%	1222.05 ± 66.74		
	2005–2009	White	62.8%	565.99 ± 23.85	216%	$p < 0.05$

Table S7. *Cont.*

States	Year	Race	Demographics	Infant Mortality (per 100,000)	Differences Compare to Black Race	Significance Compare to Black Race ($\alpha = 0.05$)
Louisiana	2005–2009	Black or African American	32.4%	1358.93 ± 146.48		
	2005–2009	White	63.7%	662.84 ± 44.34	205%	$p < 0.05$
Mississippi	2005–2009	Black or African American	37.4%	1438.27 ± 161.62		
	2005–2009	White	59.9%	707.55 ± 33.27	203%	$p < 0.05$
US	2005–2009	Asian or Pacific Islander	5.1%	462.592 ± 19.94178	275%	$p < 0.05$
	2005–2009	Black or African American	13.1%	1271.324 ± 45.90073		
	2005–2009	White	77.9%	556.168 ± 14.44635	229%	$p < 0.05$

Table S8. Comparison of the significance between race and infant mortality in the southeastern states.

Race	Significance Level									
	AL-FL	AL-GA	AL-LA	AL-MS	FL-GA	FL-LA	FL-MS	GA-LA	GA-MS	LA-MS
Asian or Pacific Islander	NA	NA	NA	NA	NS	$p < 0.05$	NA	$p < 0.05$	NA	NA
Black or African American	NS	NS	NS	NS	NS	NS	$p < 0.05$	NS	$p < 0.05$	NS
White	NS	NS	NS	NS	NS	NS	NS	NS	NS	NS

Table S9. Differences in infant mortality by maternal education in the southeastern us from 2005 to 2009.

Maternal Education	Average Infant Mortality (per 100,000) (2005–2009)					
	Alabama	Florida	Georgia	Louisiana	Mississippi	US
8th grade or less	940.17 ± 77.34 *	765.06 ± 64.63	617.83 ± 57.46	1401.98 ± 393.01 *	1309.19 ± 80.30 *	644.98 ± 25.85
9th through 12th grade with no diploma	1133.75 ± 64.76	944.30 ± 22.46	1010.00 ± 42.75	1288.84 ± 129.26 *	1630.58 ± 175.70 *	879.61 ± 59.91
High school graduate or GED completed	1065.47 ± 32.37 *	812.08 ± 17.75	884.66 ± 70.10	1115.23 ± 20.92 *	1070.48 ± 45.42 *	756.45 ± 53.72
Some college credit, but not a degree	874.29 ± 53.64 *	622.86 ± 11.29	742.85 ± 37.78	828.26 ± 11.13	880.86 ± 117.86 *	603.01 ± 7.23
College or higher	549.92 ± 101.48	480.51 ± 47.84	487.68 ± 94.06	613.62 ± 56.26	735.80 ± 11.39 *	407.96 ± 9.26

* $p < 0.05$, compared to the US.

Table S10. Comparison of the significance between maternal education and infant mortality in the southeastern states.

Maternal Education	Significance Level									
	**AL-FL**	**AL-GA**	**AL-LA**	**AL-MS**	**FL-GA**	**FL-LA**	**FL-MS**	**GA-LA**	**GA-MS**	**LA-MS**
8th grade or less	NS	$p < 0.05$	$p < 0.05$	$p < 0.05$	NS	$p < 0.05$	$p < 0.05$	$p < 0.05$	$p < 0.05$	NS
9th through 12th grade with no diploma	NS	NS	NS	$p < 0.05$	NS	$p < 0.05$	$p < 0.05$	NS	$p < 0.05$	$p < 0.05$
High school graduate or GED completed	NS	NS	NS	NS	NS	$P < 0.05$	NS	NS	NS	NS
Some college credit, but not a degree	NS	NS	NS	NS	NS	NS	NS	NS	NS	NS
College or higher	NS	NS	NS	NS	NS	NS	NS	NS	NS	NS

Table S11. Comparison of the significance between the month prenatal care began and infant mortality in the southeastern states.

Month Prenatal Care Began	Significance Level									
	**AL-FL**	**AL-GA**	**AL-LA**	**AL-MS**	**FL-GA**	**FL-LA**	**FL-MS**	**GA-LA**	**GA-MS**	**LA-MS**
No prenatal care	NS	NS	NS	$p < 0.05$	NS	NS	$p < 0.05$	NS	$p < 0.05$	NS
1st month	NS	NS	NS	NS	NS	NS	**NS**	NS	NS	NS
2nd month	NS	NS	NS	NS	NS	NS	NS	NS	NS	NS
3rd month	NS	NS	NS	NS	NS	NS	NS	NS	NS	NS
4th month	NS	NS	NS	NS	NS	NS	NS	NS	NS	NS
5th month	NS	NS	NS	NS	NS	NS	NS	NS	NS	NS
6th month	NS	NS	NS	NS	NS	NS	NS	NS	NS	NS
7th month	NS	NS	NA	NA	NS	NA	NA	NA	NA	NA

Table S12. Comparison of the significance between birth weight and infant mortality in the southeastern states.

Birth Weight (Grams)	Significance Level									
	AL-FL	AL-GA	AL-LA	AL-MS	FL-GA	FL-LA	FL-MS	GA-LA	GA-MS	LA-MS
499 or less	NS	$p < 0.05$	$p < 0.05$	NS	$p < 0.05$	$p < 0.05$	NS	$p < 0.05$	$p < 0.05$	$p < 0.05$
500–999	NS	NS	NS	NS	NS	NS	**NS**	NS	NS	$p < 0.05$
1000–1499	NS	NS	NS	NS	NS	NS	NS	NS	NS	NS
1500–1999	NS	NS	NS	NS	NS	NS	NS	NS	NS	NS
2000–2499	NS	NS	NS	NS	NS	NS	NS	NS	NS	NS
2500–2999	NS	NS	NS	NS	NS	NS	NS	NS	NS	NS
3000–3499	NS	NS	NS	NS	NS	NS	NS	NS	NS	NS
3500–3999	NS	NS	NS	NS	NS	NS	NS	NS	NS	NS
4000–4499	NA	NA	NA	NA	NS	NA	NA	NA	NS	NA

Table S13. Differences in infant mortality by the age of infant at death in the southeastern us from 2005 to 2009.

Age of Infant at Death		Average Infant Mortality (per 100,000) (2005–2009)					
		Alabama	Florida	Georgia	Louisiana	Mississippi	US
Neonatal deaths	Under 1 h	130.18 ± 7.00	99.84 ± 5.42	106.83 ± 11.94	86.74 ± 18.15	121.36 ± 21.72	96.61 ± 3.35
	1–23 h	189.53 ± 27.80	168.38 ± 8.89	184.56 ± 13.49	226.08 ± 25.63 *	233.96 ± 9.76 *	168.55 ± 4.67
	1–6 days	117.45 ± 26.23	87.37 ± 9.09	108.94 ± 8.13	104.86 ± 13.49	118.62 ± 9.90	84.19 ± 4.28
	7–27 days	138.32 ± 14.21 *	101.81 ± 9.79	118.16 ± 9.45	122.32 ± 10.64	149.59 ± 13.98 *	88.61 ± 3.72
Postneonatal deaths	28–364 days	348.32 ± 24.10 *	257.89 ± 11.85	271.01 ± 19.13 *	396.93 ± 27.36 *	416.72 ± 31.67 *	228.01 ± 5.78
Declines for neonatal mortality (%)		13.13	1.57	8.48	7.62	9.47	7.93
Declines for postneonatal mortality (%)		13.17	10.27	10.48	13.94	15.51	4.54

* $p < 0.05$, compared to the US.

Table S14. Comparison of the significance between age of infant at death and infant mortality in the southeastern states.

Age Of Infant At Death	Significance Level									
	AL-FL	AL-GA	AL-LA	AL-MS	FL-GA	FL-LA	FL-MS	GA-LA	GA-MS	LA-MS
Under 1 h	NS	NS	$p < 0.05$	NS	NS	NS	NS	NS	NS	NS
1–23 h	NS	NS	NS	$p < 0.05$	NS	$p < 0.05$	$p < 0.05$	$p < 0.05$	$p < 0.05$	NS
1–6 days	NS	NS	NS	NS	NS	NS	NS	NS	NS	NS
7–27 days	NS	NS	NS	NS	NS	NS	$P < 0.05$	NS	NS	NS
28–364 days	$p < 0.05$	$p < 0.05$	$p < 0.05$	$p < 0.05$	NS	$p < 0.05$	$p < 0.05$	$p < 0.05$	$p < 0.05$	NS

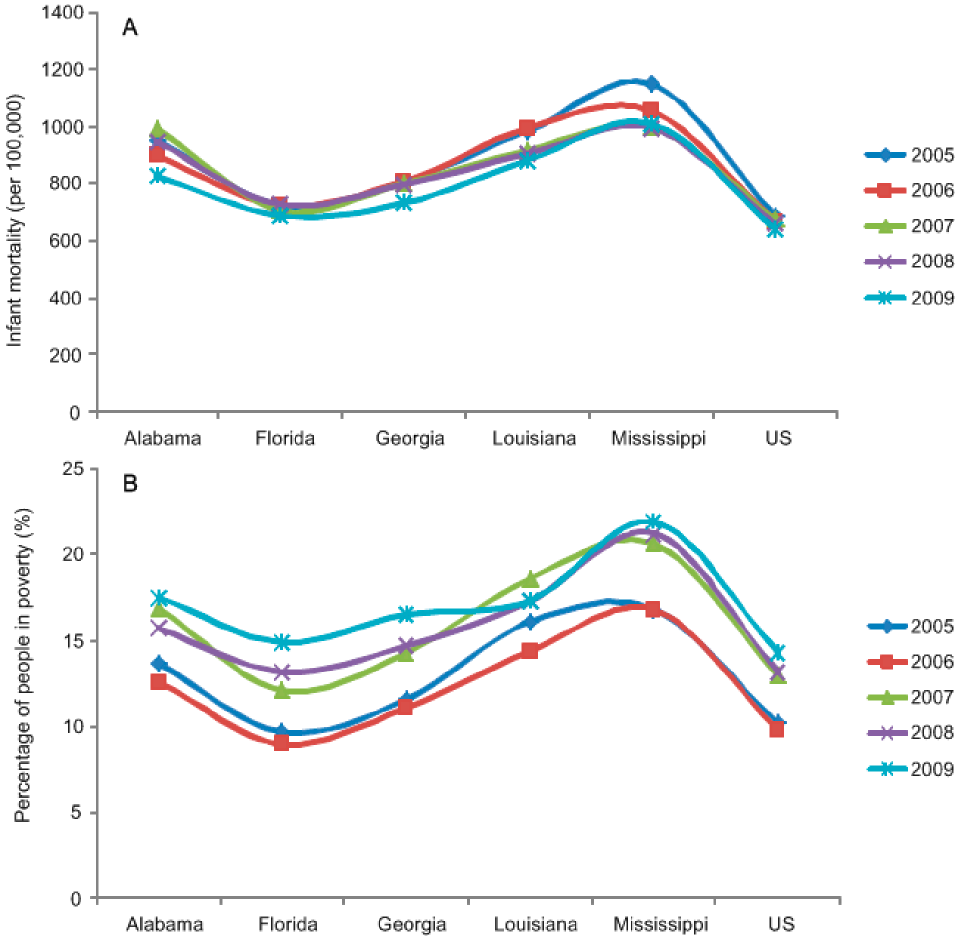

Figure S1. Trends of infant mortality (**A**) and poverty level (**B**) in the southeastern US from 2005–2009.

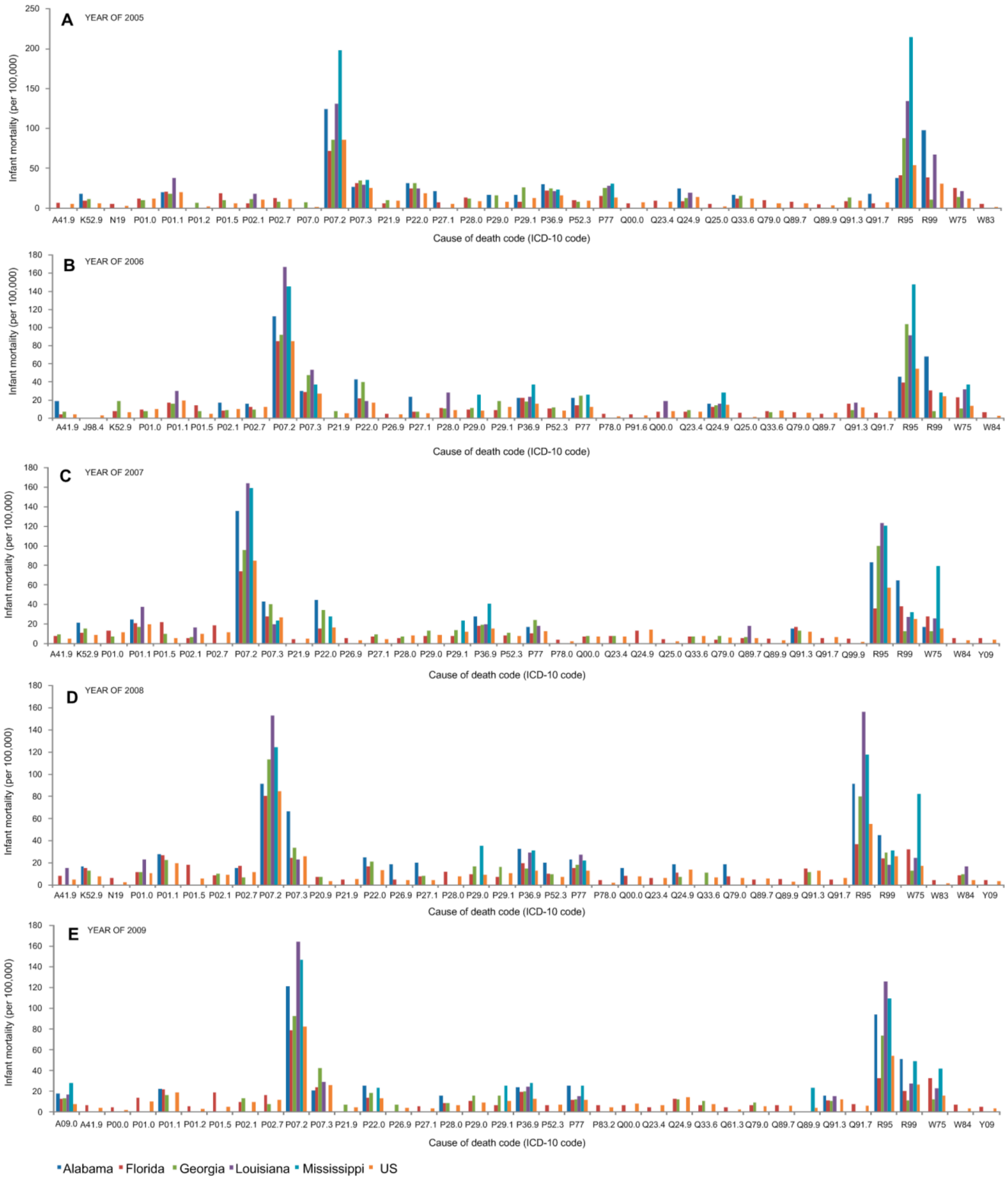

Figure S2. Causes of infant mortality in the southeastern US from 2005 (**A**) to 2009 (**E**). The six leading causes are: P07.2 (Extreme immaturity), R95 (Sudden infant death syndrome), R99 (Other ill-defined and unspecified causes of mortality), P07.3 (Other preterm infants), W75 (Accidental suffocation and strangulation in bed), P22.0 (Respiratory distress syndrome of newborn).

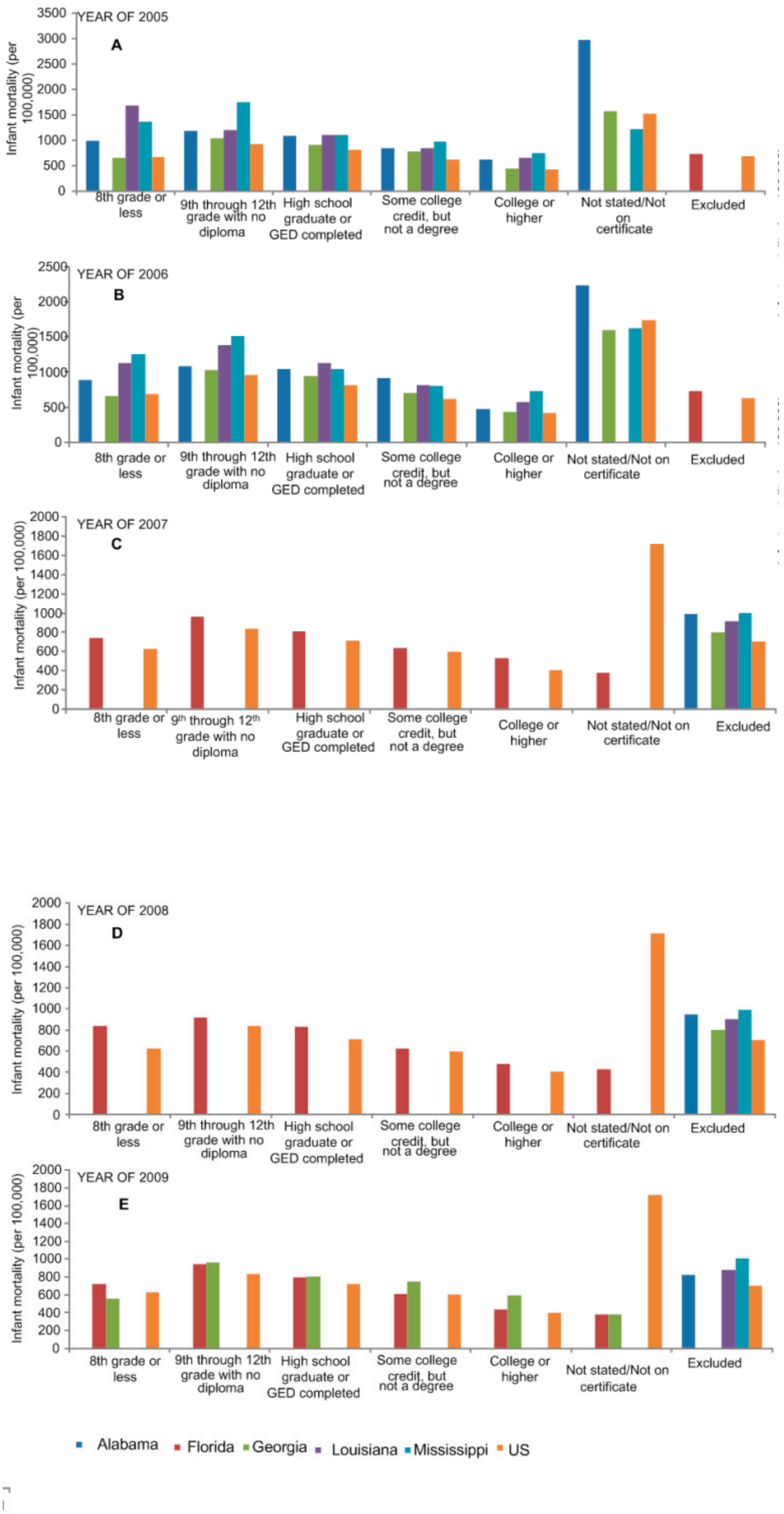

Figure S3. Correlation between infant mortality and maternal education in the southeastern US from 2005 (**A**) to 2009 (**E**).

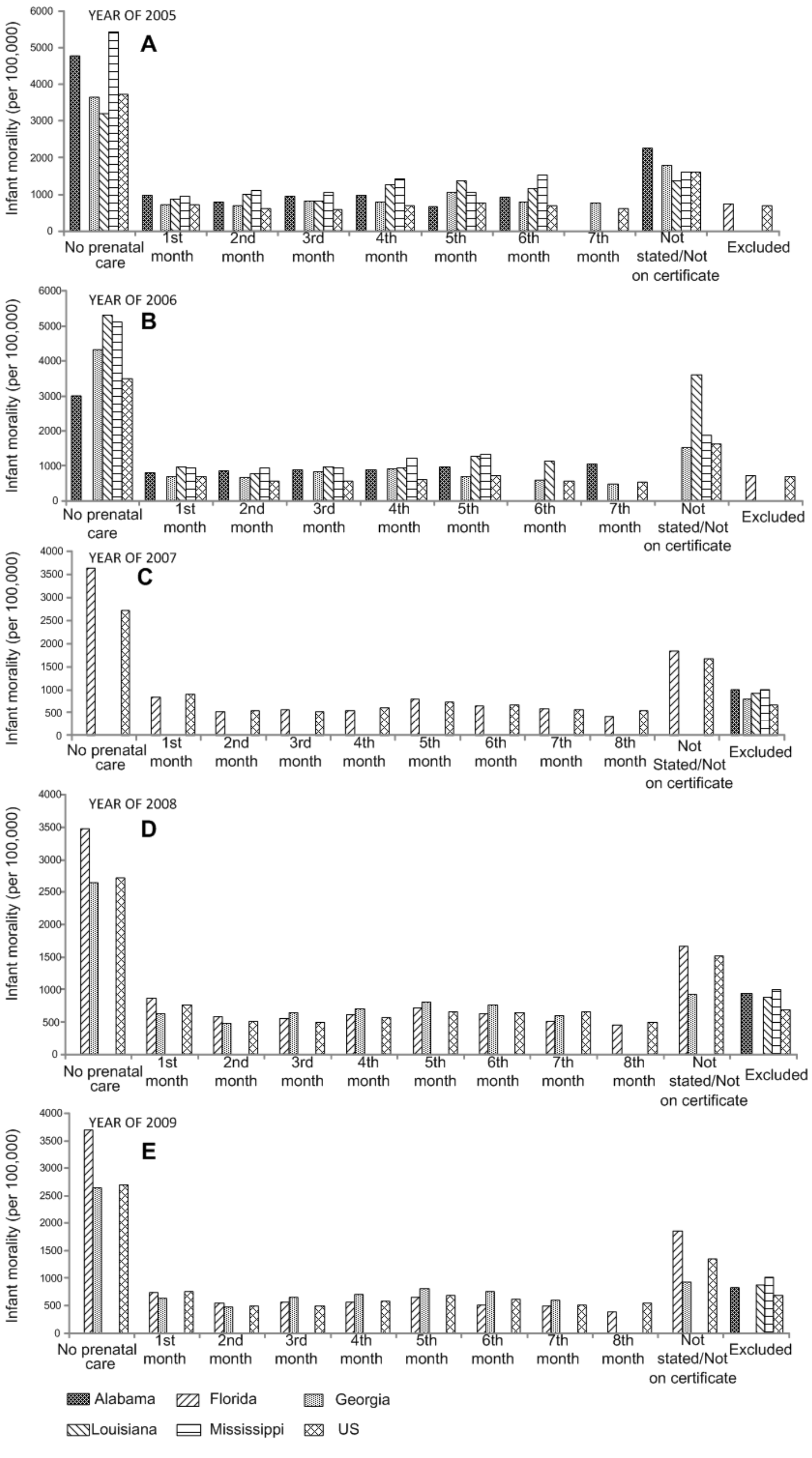

Figure S4. Analysis of variance shows the significant difference in infant mortality by the month of prenatal care began in the southeastern US from 2005 (**A**) to 2009 (**E**).

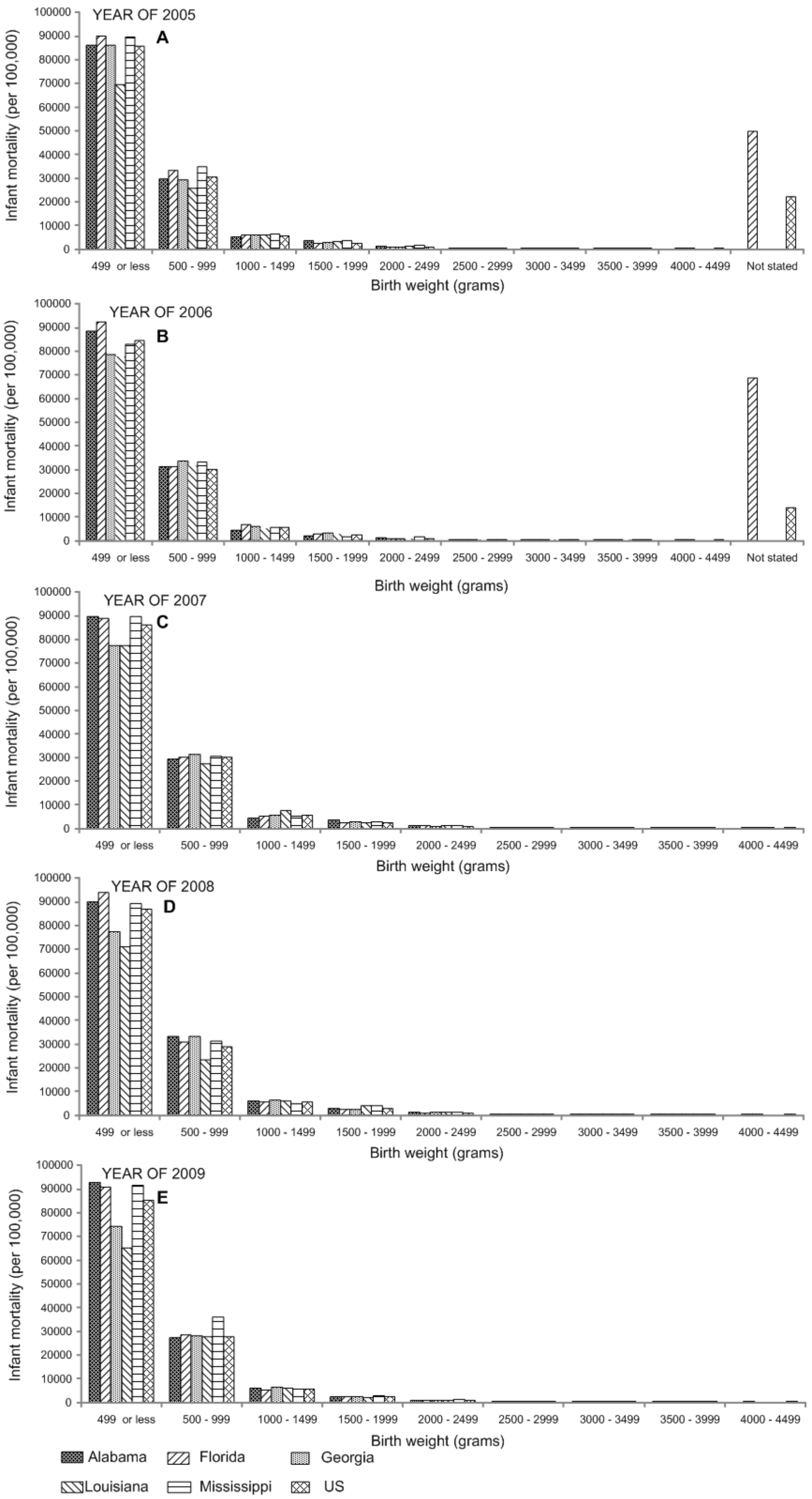

Figure S5. Analysis of variance shows the significant difference in infant mortality by the birth weight in the southeastern US from 2005 (**A**) to 2009 (**E**).

© 2015 by the authors; licensee MDPI, Basel, Switzerland. This article is an open access article distributed under the terms and conditions of the Creative Commons Attribution license (http://creativecommons.org/licenses/by/4.0/).
